# Comparison of tagging single-nucleotide polymorphism methods in association analyses

**DOI:** 10.1186/1753-6561-1-s1-s6

**Published:** 2007-12-18

**Authors:** Ellen L Goode, Brooke L Fridley, Zhifu Sun, Elizabeth J Atkinson, Alex S Nord, Shannon K McDonnell, Gail P Jarvik, Mariza de Andrade, Susan L Slager

**Affiliations:** 1 Department of Health Sciences Research, Mayo Clinic College of Medicine, 200 First Street SW, Rochester, MN 55905, USA; 2 Division of Medical Genetics, University of Washington, Box 357720, Seattle, WA 98195-7720, USA

## Abstract

Several methods to identify tagging single-nucleotide polymorphisms (SNPs) are in common use for genetic epidemiologic studies; however, there may be loss of information when using only a subset of SNPs. We sought to compare the ability of commonly used pairwise, multimarker, and haplotype-based tagging SNP selection methods to detect known associations with quantitative expression phenotypes. Using data from HapMap release 21 on unrelated Utah residents with ancestors from northern and western Europe (CEPH-Utah, CEU), we selected tagging SNPs in five chromosomal regions using ldSelect, Tagger, and TagSNPs. We found that SNP subsets did not substantially overlap, and that the use of trio data did not greatly impact SNP selection. We then tested associations between HapMap genotypes and expression phenotypes on 28 CEU individuals as part of Genetic Analysis Workshop 15. Relative to the use of all SNPs (n = 210 SNPs across all regions), most subset methods were able to detect single-SNP and haplotype associations. Generally, pairwise selection approaches worked extremely well, relative to use of all SNPs, with marked reductions in the number of SNPs required. Haplotype-based approaches, which had identified smaller SNP subsets, missed associations in some regions. We conclude that the optimal tagging SNP method depends on the true model of the genetic association (i.e., whether a SNP or haplotype is responsible); unfortunately, this is often unknown at the time of SNP selection. Additional evaluations using empirical and simulated data are needed.

## Background

Development and application of methods using linkage-disequilibrium (LD) for single-nucleotide polymorphism (SNP) selection has empowered genetic epidemiologic studies. Tagging SNP selection methods capitalize on the high levels of LD in much of the genome and aim to capture all of the common variation. SNP redundancy can be reduced, allowing for improved information/coverage within the constraints of a fixed budget. Three classes of tagging SNP methods have the following aims: 1) correlate each SNP of interest with a genotyped SNP (pairwise methods), 2) correlate each SNP of interest with a genotyped SNP or a combination of genotyped SNPs (multimarker methods), or 3) explain each haplotype of interest using a set of genotyped SNPs (haplotype-based methods). Investigators commonly select tagging SNPs using data from public projects [1] or a subset of study participants, then genotype only the SNP subset in the larger study population [2,3].

Tagging SNP selection is implemented in commonly used, publicly available software packages that assess data from unrelated individuals (founders) or small families (trios). ldSelect [4] performs pairwise selection using a binning algorithm, Tagger [5] selects SNPs using pairwise and multimarker methods and allows for inclusion of trio data to reduce phase uncertainty, and TagSNPs v. 2.0-beta [6] implements pairwise, multimarker, and haplotype methods allowing for the inclusion of trio data.

We used these tagging SNP selection methods in genomic regions known to harbor associations with quantitative phenotypes [7]. We sought to assess whether (and to what degree) associations would have been detected if SNP subsets, rather than all SNPs, had been used. Previous simulated [8,9] and family-based [10,11] analyses suggest that empirical tagging SNP assessment in the context of association testing is needed. Here, we examine associations from analysis of >770,000 HapMap Phase I genotypes and ~1,000 expression phenotypes in 57 unrelated Utah residents with ancestors from northern and western Europe (CEU) [7]. We conducted a pilot study using a subset of samples with HapMap Phase II genotypes and contributed expression phenotypes as part of Genetic Analysis Workshop 15 (GAW15) [12].

## Methods

Selection of regions to study was based on genetic associations with lymphocyte expression values reported by Cheung et al. [7]. Using linear regression and limiting the data to 28 individuals with both HapMap and GAW15 data (described in more detail below), excluding rs535088 (genotypes not available) and PSPHL (not uniquely mapped), we reassessed the ten most statistically significant genotype-phenotype pairs reported. Regions containing the five strongest associations (Table 1) were defined as 5 kb surrounding the previously reported SNPs and the nearby (*cis*) gene of interest.

**Table 1:** Chromosomal regions^a^

Chr (Mb)	Size (kb)	*N *SNPs^b^	Mean r^2^	Protein (Probe set)	Original SNP^c^	Cheung et al. [7] *p*-value	Current *p*-value^d^
5 (96)	68	72	0.65	LRAP (219759_at)	rs2762	2.0 × 10^-19^	8.0 × 0^-11^
6 (32)	90	52	0.20	HLA-DRB2 (209480_at)	rs6928482	6.5 × 10^-11^	3.8 × 10^-7^
20 (33)	49	44	0.58	CPNE1 (206918_s_at)	rs6060535	8.4 × 10^-13^	1.3 × 10^-7^
20 (36)	25	16	0.73	AA827892 (65588_at)	rs788350	3.7 × 10^-15^	1.6 × 10^-5^
21 (44)	42	26	0.40	CSTB (201201_at)	rs880987	2.5 × 10^-12^	6.5 × 10^-6^

^a^Regions defined as ± 5 kb surrounding original SNP and *cis *gene.

^b^Based on MAF > 0.05 in CEU HapMap release 21.

^c^rs Identification number shown is that reported by Cheung et al. based on 57 CEU founders [7].

^d^Current *p*-value based on 28 CEU founders.

Tagging SNP selection within these regions utilized HapMap release 21 CEU genotype data (60 founders or 30 trios) with MAF (or haplotype frequency) ≥ 0.05 and no quality control exclusions [13]. These parameters were chosen on the basis of common use in genetic association studies. From starting sets of "All SNPs", pairwise methods used a threshold of *r*^2 ^≥ 0.8 between unassayed and assayed SNPs among founders ("ldSelect", "TagSNPs-Rspair") or trios ("TagSNPs-Rspair-trios", "Tagger-pairwise"); multimarker methods used *R*_*s*_^2 ^≥ 0.8 (or LOD > 3.0) between unassayed SNPs and combinations of up to three assayed SNPs among founders ("TagSNPs-Rs") or trios ("TagSNPs-Rs-trios", "Tagger-multimarker"); haplotype-based methods used *R*_*h*_^2 ^≥ 0.8 between haplotypes and assayed SNPs among founders ("TagSNPs-Rh") or trios ("TagSNPs-Rh-trios").

Association testing was performed on 28 unrelated CEU individuals included in both HapMap and GAW15 datasets (IDs available upon request) [1,13]. We used genotypes from HapMap release 21 (coded as 0, 1, and 2) and phenotypes from GAW15 (log_2_-transformed Affymetrix global-normalized lymphocyte expression values [14]). Single-SNP association testing used linear regression [7]. Haplotype association testing used the Splus library *HaploStat *[15] excluding haplotypes with estimated n < 5. Haplotypes were defined across each region (haplo.score) as well as by sliding three-SNP windows (haplo.score.slide) [15].

## Results

**Figure 1:**
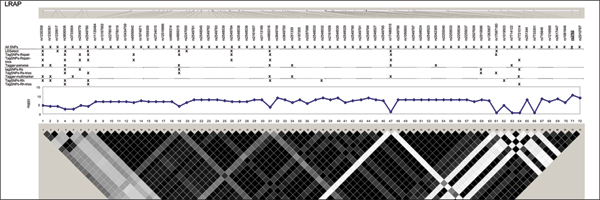
**SNPs, single-SNP associations, and LD for LRAP**. Underline, original association; Haploview 3.32 plotted *r*^2 ^(white, 0; black, 1) in 60 CEU samples.

**Figure 2:**
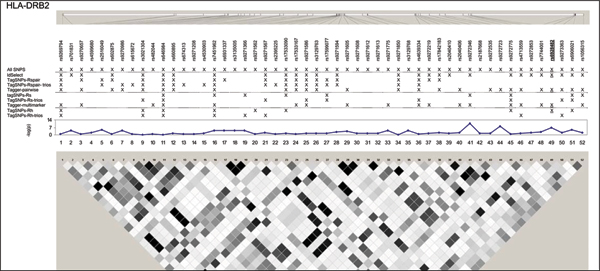
**SNPs, single-SNP associations, and LD for HLA-DRB2**. Underline, original association; Haploview 3.32 plotted *r*^2 ^(white, 0; black, 1) in 60 CEU samples.

**Figure 3:**
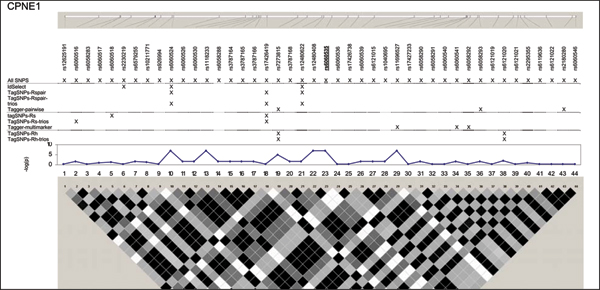
**SNPs, single-SNP associations, and LD for CPNE1**. Underline, original association; Haploview 3.32 plotted *r*^2 ^(white, 0; black, 1) in 60 CEU samples.

**Figure 4:**
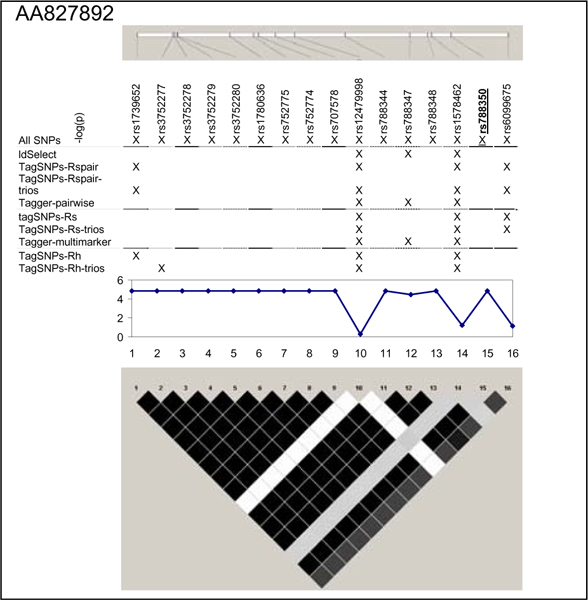
**SNPs, single-SNP associations, and LD for AA827892**. Underline, original association; Haploview 3.32 plotted *r*^2 ^(white, 0; black, 1) in 60 CEU samples.

**Figure 5:**
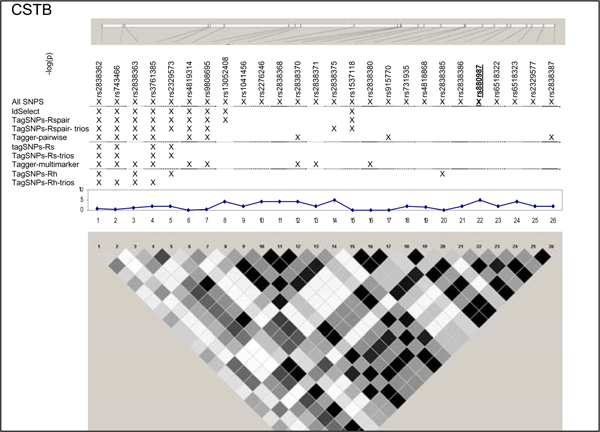
**SNPs, single-SNP associations, and LD for CSTB**. Underline, original association; Haploview 3.32 plotted *r*^2 ^(white, 0; black, 1) in 60 CEU samples.

We examined five regions known to harbor genetic associations in a small, well characterized sample [7]. SNPs in these chromosomes 5, 6, 20, and 21 regions were associated with lymphocyte expression levels of proteins (LRAP, HLA-DRB2, CPNE1, AA827892, and CSTB) encoded by nearby genes (Table 1). The HapMap project genotyped a total of 210 SNPs (MAF ≥ 0.05 in 60 CEU samples) (Figure 1, 2, 3, 4, 5). The LRAP region included the most HapMap SNPs (n = 72, Table 1) and had strong linkage disequilibrium (LD); the HLA-DRB2 region had a large number of SNPs and low LD; the AA827892 region included only 16 SNPs in strong LD; and the CPNE1 and CSTB regions were of intermediate size with modest/variable LD. Single-SNP association testing in 28 phenotyped individuals yielded *p*-values < 10^-6 ^in each region (Figure 1, 2, 3, 4, 5). Across regions of strong LD, consistent associations were seen (i.e., nearly identical -log_10_(*p*-values)); independent SNPs yielded unique results (Figure 4).

Nine subsets of tagging SNPs were identified within each region (Figure 1, 2, 3, 4, 5). In regions with lower LD (HLA-DRB2 and CSTB), more markers were generally required and selected SNPs were less consistent across methods. This may be because there are many possible haplotypes, and haplotype-based methods may thus estimate varying number and frequency of the haplotypes to tag. In regions with high-LD, there was also lack of consistency across methods. For example, in the AA827892 region, SNPs 10 and 14 are independent and selected by all methods, yet SNPs 1–9 and 11–13 are in high LD and methods vary in which they select (Figure 4). There were surprising discrepancies in SNP selection across methods that used an identical algorithm (e.g., ldSelect and TagSNPs-Rspair); we attribute this to differences in rounding LD measures. Generally, SNP subsets overlapped among pairwise methods (HLA-DRB2, Figure 2), among haplotype-based methods (CPNE1, Figure 3), among TagSNPs methods with trios and founders (LRAP, Figure 1), and among Tagger pairwise and multimarker methods (CSTB, Figure 5).

We then assessed whether subsets of tagging SNPs detected the strong association signals observed when all SNPs were studied (Table 1). The minimum single-SNP association *p*-values identified by each subset within each region are provided in Table 2. Single-SNP results in each region were strongest using "All SNPs", but were comparable in SNP subsets that included the strongest SNP or a SNP in strong LD with the strongest SNP (e.g., SNP 10, 13, and 18 in the CPNE1 region; Figure 1, 2, 3, 4, 5). Although all methods identified HLA-DRB2 associations, there was great variation in *p*-values, most likely due to one particularly strong SNP association (SNP 41) and low LD (except with SNP 44). Multimarker SNP selection methods implemented in TagSNPs (but not Tagger) failed to detect associations with CPNE1 or AA827892 (selected SNPs, e.g., AA827892 SNP 16, were not in LD with associated SNPs) (*p *> 0.01; Figure 1, 2, 3, 4, 5; Table 2).

**Table 2:** Single-SNP association results^a^

	LRAP		HLA-DRB2		CPNE1		AA827892		CSTB	
					
	*N*^b^	min(*p*)^c^	*N*	min(*p*)	*N*	min(*p*)	*N*	min(*p*)	*N*	min(*p*)
All SNPs	72	**8.0 × 10**^-11^	52	**3.4 × 10**^-11^	44	**1.3 × 10**^-7^	16	**1.5 × 10**^-5^	26	**6.5 × 10**^-6^
										
ldSelect	9	**3.6 × 10**^-7^	28	**3.4 × 10**^-11^	3	**1.3 × 10**^-7^	3	**3.7 × 10**^-5^	9	**5.4 × 10**^-5^
TagSNPs-Rspair	10	**1.3 × 10**^-7^	20	**1.8 × 10**^-5^	3	**1.3 × 10**^-7^	4	**1.5 × 10**^-5^	9	**5.4 × 10**^-5^
TagSNPs-Rspair-trios	10	**1.3 × 10**^-7^	20	**1.8 × 10**^-5^	3	**1.3 × 10**^-7^	4	**1.5 × 10**^-5^	9	**6.5 × 10**^-6^
Tagger-pairwise	9	**2.2 × 10**^-8^	26	**1.2 × 10**^-8^	3	**1.2 × 10**^-5^	3	**3.7 × 10**^-5^	9	**4.8 × 10**^-5^
										
TagSNPs-Rs	5	**1.1 × 10**^-8^	9	**3.4 × 10**^-11^	2	3.2 × 10^-2^	3	5.9 × 10^-2^	4	**9.9 × 10**^-3^
TagSNPs-Rs-trios	5	**1.1 × 10**^-8^	9	**1.4 × 10**^-5^	2	2.9 × 10^-2^	3	5.9 × 10^-2^	4	**9.9 × 10**^-3^
Tagger-multimarker	7	**3.6 × 10**^-7^	18	**3.4 × 10**^-11^	3	**1.3 × 10**^-7^	3	**3.7 × 10**^-5^	9	**4.8 × 10**^-5^
										
TagSNPs-Rh	7	**2.8 × 10**^-9^	9	**3.8 × 10**^-7^	2	**1.2 × 10**^-5^	3	**1.5 × 10**^-5^	4	**9.9 × 10**^-3^
TagSNPs-Rh-trios	6	**3.6 × 10**^-7^	8	**5.1 × 10**^-5^	2	**1.2 × 10**^-5^	3	**1.5 × 10**^-5^	4	1.5 × 10^-2^

^a^Subset methods sorted into pairwise, multimarker, and haplotype-based methods.

^b^N, number of SNPs.

^c^Bold, <0.01.

Although regions were initially chosen on the basis of observed single-SNP associations, we also assessed haplotype associations. Results considering all SNPs in each set (global *p*-value), and sliding windows of three-SNP haplotypes (minimum global *p*-value) are shown in Table 3. In all regions using "All SNPs", at least one three-SNP haplotype was associated at *p *< 0.01; but only the LRAP, CPNE1, and CSTB regions yielded global results significant at this level (Table 3). Comparing across subsets, note that set-haplotype analyses are comparable in terms of number of tests, while three-SNP haplotype analyses are comparable in terms of degrees of freedom. There was general consistency in results across methods for LRAP and AA827892 (regions with strongest LD); however, no subsets detected the strongest three-marker haplotype association for AA827892. There was also consistency in haplotype association results in the HLA-DRB2 region (with low LD); global *p*-values oscillated around 0.01. Haplotype-based SNP selection methods (TagSNPs-Rh-trios), which selected only two tagging SNPs, failed to detect the CPNE1 haplotype association observed by other methods (Table 3). Multimarker SNP selection methods implemented in TagSNPs (but not Tagger) failed to detect CSTB haplotype associations.

**Table 3:** Haplotype association results^a^

	LRAP		HLA-DRB2		CPNE1		AA827892		CSTB	
					
	*p*-set/df^b^	min(*p*)^c^	*p*-set/df	min(*p*)	*p*-set/df	min(*p*)	*p*-set/df	min(*p*)	*p*-set/df	min(*p*)
All SNPs	**1.8 × 10**^-3^**/5**^d^	**1.3 × 10**^-5^	1.6 × 10^-2^/3	**5.1 × 10**^-5^	**3.4 × 10**^-3^**/2**	**7.1 × 10**^-5^	2.1 × 10^-1^/2	**1.8 × 10**^-4^	**2.1 × 10**^-4^**/2**	**3.3 × 10**^-4^
										
ldSelect	**7.5 × 10**^-4^**/4**	**1.7 × 10**^-4^	**8.5 × 10**^-3^**/4**	**5.1 × 10**^-5^	**7.1 × 10**^-5^**/2**	**7.1 × 10**^-5^	5.2 × 10^-2^/2	5.2 × 10^-2^	**1.8 × 10**^-4^**/2**	**2.3 × 10**^-3^
TagSNPs-Rspair	**1.4 × 10**^-3^**/5**	**3.9 × 10**^-5^	2.1 × 10^-2^/3	**5.6 × 10**^-5^	**7.1 × 10**^-5^**/2**	**7.1 × 10**^-5^	2.1 × 10^-1^/2	5.2 × 10^-2^	**1.8 × 10**^-4^**/2**	**3.1 × 10**^-3^
TagSNPs-Rspair-trios	**1.4 × 10**^-3^**/5**	**3.9 × 10**^-5^	2.1 × 10^-2^/3	**2.3 × 10**^-4^	**7.1 × 10**^-5^**/2**	**7.1 × 10**^-5^	2.1 × 10^-1^/2	5.2 × 10^-2^	**2.1 × 10**^-4^**/2**	**1.1 × 10**^-3^
Tagger-pairwise	**3.3 × 10**^-4^**/4**	**1.7 × 10**^-5^	9.5 × 10^-3^/4	**5.1 × 10**^-5^	**7.1 × 10**^-5^**/2**	**7.1 × 10**^-5^	5.2 × 10^-2^/2	5.2 × 10^-2^	**1.8 × 10**^-4^**/2**	**3.0 × 10**^-3^
										
TagSNPs-Rs	**4.0 × 10**^-4^**/3**	**1.7 × 10**^-4^	4.1 × 10^-2^/3	**1.5 **× **10**^-5^	**1.1 **× **10**^-4^**/2**	**1.1 **× **10**^-4^	1.7 × 10^-1^/2	1.7 × 10^-1^	5.0 × 10^-1^/4	2.7 × 10^-2^
TagSNPs-Rs-trios	**1.2 × 10**^-3^**/4**	**1.6 × 10**^-4^	**1.3 **× **10**^-3^**/4**	**5.7 **× **10**^-4^	**7.1 **× **10**^-5^**/2**	**7.1 **× **10**^-5^	1.7 × 10^-1^/2	1.7 × 10^-1^	5.0 × 10^-1^/4	2.7 × 10^-2^
Tagger-multimarker	**3.3 × 10**^-4^**/4**	**5.7 × 10**^-5^	1.7 × 10^-2^/3	**5.7 **× **10**^-5^	**7.1 **× **10**^-5^**/2**	**7.1 **× **10**^-5^	5.2 × 10^-2^/2	5.2 × 10^-2^	**1.8 **× **10**^-4^**/2**	**1.3 **× **10**^-3^
										
TagSNPs-Rh	**8.7 × 10**^-4^**/4**	**2.0 × 10**^-4^	**2.4 **× **10**^-3^**/2**	**5.1 **× **10**^-4^	1.3 × 10^-2^**/1**	1.3 × 10^-2^	5.2 × 10^-2^/2	5.2 × 10^-2^	**5.5 **× **10**^-4^**/3**	**3.2 **× **10**^-3^
TagSNPs-Rh-trios	**6.4 × 10**^-4^**/3**	**1.4 × 10**^-4^	2.8 × 10^-2^/4	**4.1 **× **10**^-4^	1.3 × 10^-2^**/1**	1.3 × 10^-2^	5.2 × 10^-2^/2	5.2 × 10^-2^	**1.2 **× **10**^-4^**/2**	**8.4 **× **10**^-3^

^a^Subset methods are sorted into pairwise, multimarker, and haplotype-based methods.

^b^*p*-set/df, global score test considering entire set.

^c^min(*p*), smallest *p*-value considering three-SNP haplotypes across set.

^d^Bold, <0.01.

**Figure 6:**
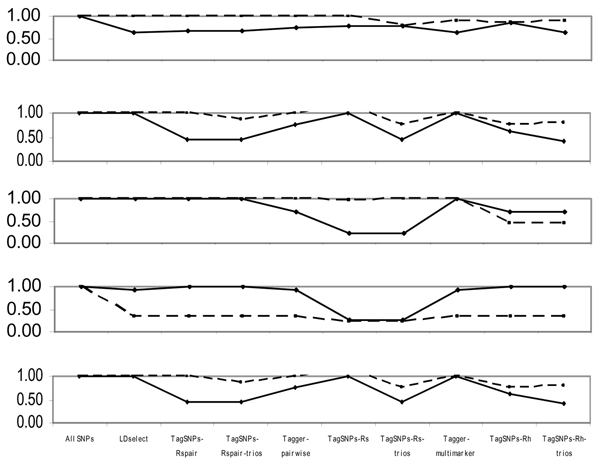
**Relative signal strength**. [-log(min-*p*-Subset)]/[-log(min-*p*-All-SNPs)]; solid line, single-SNP; dashed line, 3-SNP haplotype.

Figure 6 summarizes relative signals for associations across SNP subsets as the ratio of [-log(minimum *p*-value using subset)] to [-log(minimum *p*-value using all SNPs)]. Generally, haplotype-based selection methods and methods in TagSNPs "missed" more single-SNP and haplotype associations than other methods (Figure 6).

## Discussion

Our ability to combine HapMap genotype data with GAW15 phenotype data provided a unique opportunity to assess chromosomal regions harboring known genetic associations in CEU samples. Although only a small pilot study, we explored whether these associations would have been detected if genotyping had been limited to tagging SNPs. The current analysis has advantages over other reported methods in that we focused on association testing, particular commonly used statistical tools, and use of HapMap data.

We make several observations. There was lack of consistency across selected SNP sets whether or not LD was present. Inclusion of trio data did not generally impact SNP selection. For the majority of regions, pairwise approaches worked well, relative to use of all SNPs, with marked reductions in the number of SNPs required. Methods reducing the number of SNPs over pairwise methods (e.g., multimarker methods) may lead to more missed signals, particularly in haplotype association testing. The program TagSNPs did not offer particular advantages over ldSelect or Tagger in terms of number of SNPs chosen or associations detected. Regardless of the method used, typing additional markers in areas of signal may improve signal strength and localization.

The current work suggests that empirical assessment of a larger data set and simulated data addressing a range of genetic models would allow for more precise comparison of approaches. Consideration of coverage, rather than signal strength, and examination of our assumption that signals detected in each region were due to a common underlying genetic cause could further inform comparisons. Additional issues include cost efficiency, transferability of tagging SNPs, and the role of bioinformatics.

## Conclusion

The optimal tagging SNP method to use will depend on the true genetic model of the association. Pairwise or multimarker methods are optimal if the discovery SNP set contains the causal SNP (or a SNP in strong LD with causal SNP), while haplotype-based methods are optimal if the discovery SNP set defines a haplotype carrying the causal allele. Unfortunately, it is seldom known during the SNP selection phase of studies whether a SNP or a haplotype defines an association. Thus, critical assessment of the utility of available SNP selection methods under a variety of conditions is essential.

## Competing interests

The author(s) declare that they have no competing interests.
